# Detect accessible chromatin using ATAC-sequencing, from principle to applications

**DOI:** 10.1186/s41065-019-0105-9

**Published:** 2019-08-15

**Authors:** Yuanyuan Sun, Nan Miao, Tao Sun

**Affiliations:** Center for Precision Medicine, School of Medicine and School of Biomedical Sciences, Huaqiao University, 668 Jimei Road, Xiamen, 361021 Fujian China

**Keywords:** Chromatin accessibility, Tn5 transposase, ATAC-seq, Promoter, Enhancer

## Abstract

**Background:**

Chromatin accessibility is crucial for gene expression regulation in specific cells and in multiple biological processes. Assay for Transposase Accessible Chromatin with high-throughput sequencing (ATAC-seq) is an effective way to reveal chromatin accessibility at a genome-wide level. Through ATAC-seq, produced reads from a small number of cells reflect accessible regions that correspond to nucleosome positioning and transcription factor binding sites, due to probing hyperactive Tn5 transposase to DNA sequence.

**Conclusion:**

In this review, we summarize both principle and features of ATAC-seq, highlight its applications in basic and clinical research. ATAC-seq has generated comprehensive chromatin accessible maps, and is becoming a powerful tool to understand dynamic gene expression regulation in stem cells, early embryos and tumors.

## Background

In eukaryotic cells, chromatin is a basic hereditary unit, which consists of DNA, histone proteins and other genetic materials, and regulates cell type-specific gene expression [1, 2]. Chromatin, as a dynamic nuclear structure, is transcriptionally active in the interphase, and is relatively inactive in the metaphase in a cell cycle [3]. Regulation of transcription is a dynamic interaction between chromatin structure and recruitment of numerous transcription factors to the enhancers, upstream activator sequences, and proximal promoter elements. These transcription factors recruit RNA polymerase to the core promoter for productive transcription [4].

**Fig. 1:**
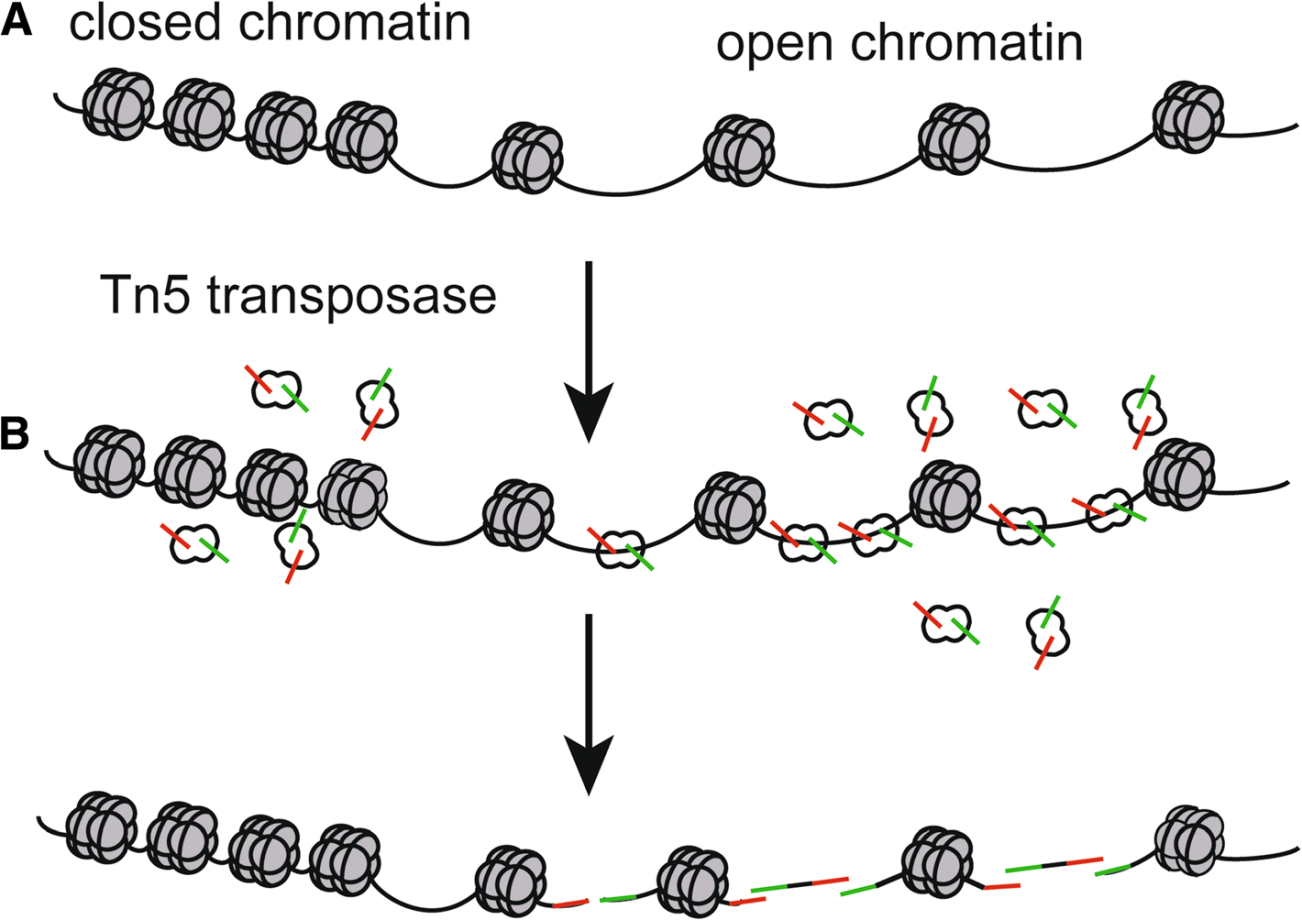
The mechanism of identifying chromatin accessibility using the Tn5 transposase. **a** Open and closed status of chromatin. **b** When the chromatin accessibility is increased, the Tn5 transposase transpose in the open chromatin more often than in the inaccessible chromatin. Then the Tn5 transposase cuts the open chromatin and tags the adaptors to it to generate DNA fragments. The green symbol represents “adapter 1” of the Tn5 transposase, while the red symbol represents “adapter 2” of theTn5 transposase

In general, the regulatory elements selectively localize in the accessible chromatin, which is crucial to transcriptional regulation [5]. Although transcription factor occupancy is not necessarily positively correlated with chromatin accessibility [6], the maintenance of accessible chromatin configurations requires binding of transcription factors to activate target genes [7] (Fig. 1a). On the other hand, condensed chromatin, known as closed chromatin, restricts binding of transcription factors and transcriptional regulators to the promoter and/or enhancer, which results in gene silencing [8–10] (Fig. 1a). Moreover, chromatin accessibility is a substantial part of epigenetic regulation, which is marked by DNA methylation and histone modification [11]. Environmental pollution factors, such as polycyclic aromatic hydrocarbons (PAHs), can affect DNA methylation [12, 13]. Therefore, chromatin accessibility, which can be modified by some environmental and pathogenic factors, indicates positions of nucleosome and regulatory regions such as enhancers, reflects precise regulations of cell behaviors, and implies dynamic physiological processes or disease conditions [14–19].

Changes of the chromatin structure occur at specific ribozyme accessibility sites that are associated with transcriptional initiation or some specific DNA structures such as specific hypersensitive sites [20]. These sites in DNA double strands can be digested by DNA enzyme I (DNase I), which reflects the accessibility of chromatin [21]. The hypersensitive sites, mostly in the promoter region, are related to gene expression [22]. To reveal accessible chromatin regions in real time and at a genome-wide level, a method named Assay for Transposase Accessible Chromatin with high-throughput sequencing (ATAC-seq) was developed and quickly applied in various studies of gene expression. ATAC-seq utilizes the Tn5 transposase and the transposable DNA as adapters, which allows the adapter introduced into the accessible chromatin [19].

Here we summarize the principle of the ATAC-seq method, highlight its usage in understanding basic transcription programs in specific cell types of humans and mice, and in revealing genetic reasoning of human diseases.

## Summary of the ATAC-seq method

### Principle and procedures of ATAC-seq

**Fig. 2:**
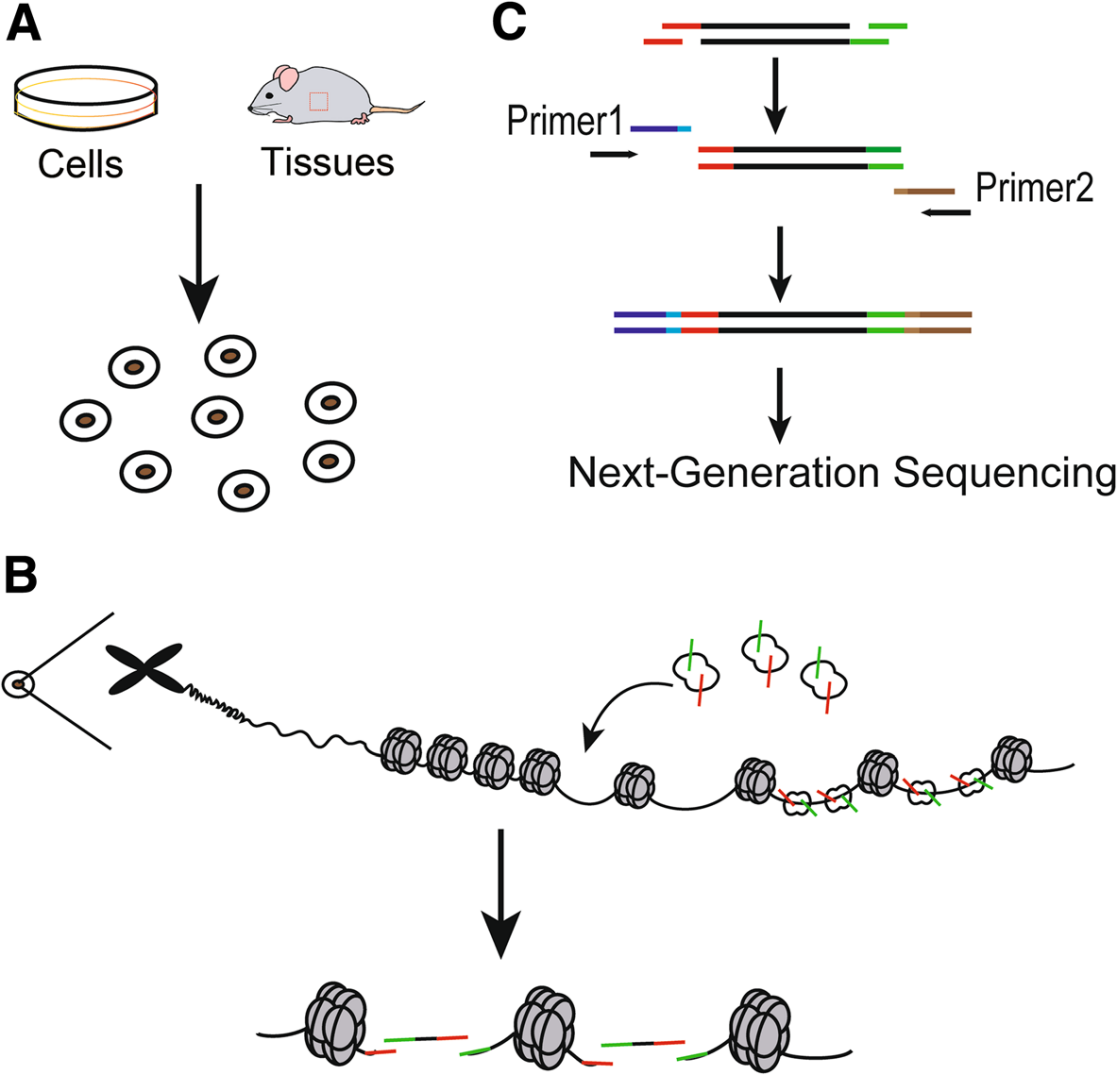
The major procedures of ATAC-seq. **a** Nuclei preparation: target cells are lysed in the lysis buffer to collect nuclei. **b** Transposase reaction: adding the Tn5 transposase to tag the genomic DNA. The green symbol represents “adapter 1” of the Tn5 transposase, while the red symbol represents “adapter 2” of theTn5 transposase. **c** PCR amplification: PCR primer-1 and -2 are used to generate library for sequencing. Primer-1 and -2 are two universal PCR primers, which capture fragments with special length and add barcodes appropriate for the next generation sequencing

ATAC-seq is an innovative epigenetic technology, which is a method for mapping chromatin accessibility by probing hyperactive Tn5 transposase to DNA sequence at a genome-wide level [23] (Fig. 1b). DNA transposon is a phenomenon that transfers DNA sequence from one region of chromosome to another, which is assisted by DNA transposase [24]. DNA transposon requires that the chromatin at the insertion site is open, and the transposase carrying known DNA sequence tags needs to be artificially added to the nucleus, and then the open chromatin can be identified by using labels of known sequences to construct a library for sequencing [23]. At present, the most commonly used transposase is the Tn5 transposase, which can transpose in the accessible chromatin more often than in the inaccessible chromatin. The Tn5 transposase acts as a probe for measuring chromatin accessibility at the genome-wide level through the “cut and paste” mechanism, and the transposon can simultaneously fragment and tag the unprotected regions of DNA with sequencing adapters [25, 26] (Fig. 1b and Fig. 2).

The construction of ATAC-seq library consists of three steps: nuclei preparation, transposition and amplification [19] (Fig. 2). Firstly, tissues or cells for examination are suspended into intact, homogenous single cells, which are subsequently incubated in the lysis buffer to generate crude nuclei (Fig. 2a). Secondly, the re-suspended nuclei are incubated in the transposition reaction mix to yield DNA fragment (Fig. 2b). Finally, transposed DNA is amplified to generate libraries for sequencing (Fig. 2c). The reaction of transposable enzyme to the chromatin of the sample is the key step of the ATAC experiment [27].

Quality control of the ATAC-seq library should be performed prior to sequencing to guarantee that the library concentration reaches the sequencing criteria. After library sequencing, raw reads are collected through sequencing the qualified library. After filtering data through sequencing data quality assessment, clean reads are further obtained by evaluating sequencing quality and summarizing data production [18, 28, 29]. After removing adapter sequences and low quality reads, high-quality reads about 150 nucleotides (nts) in length are processed for further analysis [30]. The peak calling reads are mapped to the reference genome and accessible chromatin regions, such as promoters, enhancers and insulators [31–33]. A series of detailed analysis can be further conducted, such as ascertaining distribution of reads across the whole genome, determining distribution of the peak length, functional analysis of genes with identified peaks, distribution of peaks on functional elements of genes, and analysis of differential peaks among samples [34, 35].

### Advantages of ATAC-seq

The ATAC-seq method was first developed as an alternative or supplement to sequencing of Micrococcal Nuclease sensitive sites (MNase-seq), Formaldehyde-Assisted Isolation of Regulatory Elements (FAIRE-seq), and Deoxyribonuclease I hypersensitive sites sequencing (DNase-seq) [36–38] (Table 1). For the MNase-seq and DNase-seq, when the aggregation of DNA and histone is decreased, the unprotected DNA is exposed and cut by DNA enzymes such as MNase and DNase [39, 40]. The accessible chromatin regions are recognized by sequencing the cleaved DNA fragments and comparing the sequence reads to the reference genome [39, 41, 42]. Drawbacks of these two methods are time-consuming and poor repeatability [38, 43, 44]. The FAIRE-seq uses formaldehyde immobilization, phenol chloroform extraction and separation to obtain exposed DNA. However, its background is high, and sequencing signal-to-noise ratio is low [45–47].

**Table 1:** Comparison of several sequencing methods

Methods	MNase-seq	DNase-seq	FAIRE-seq	ATAC-seq
Cell status	Any state of cells	Any state of cells	Any state of cells	Fresh cells or slowly cooled cryopreserved cells
Principle	MNase digests DNA which is not protected by protein or nucleosome on chromatin.	DNAase I preferentially excises DNA sequence without nucleosomes.	Separation of naked DNA based on formaldehyde fixation and phenol-chloroform extraction	Tn5 transpoase inserts the DNA sequence without protein or nucleosome protection and excises it.
Target regions	Focus on nucleosome localization	Accessible chromatin regions, focusing on transcription factor binding sites	Accessible chromatin regions	Accessible chromatin regions in genome-wide, including transcription factors, histone modifications.
Specific features	1. A large number of cells as input materials; 2. The quantity of enzyme needs to be accurate; 3. Localization of the entire nucleosome and inactive regulatory region; 4. Detecting inactive regions by degrading active regions; 5. Standard analysis requires 150-200 M reads.	1. A large number of cells as input materials; 2. The process of sample preparation is complicated; 3. The quantity of enzyme needs to be accurate; 4. Standard analysis requires 20-50 M reads.	1. Low signal-to-noise ratio makes data analysis difficult; 2. Results depend heavily on formaldehyde fixation; 3. Standard analysis requires 20-50 M reads.	1. A lower number of cells as input materials; 2. Standard analysis requires 20-50 M reads through reducing sequencing depth; 3. Conveniently obtain accessible chromatin regions in genome-wide; 4. Mitochondrial data has an effect on the accuracy of the results.
Time	2–3 days	2–3 days	3–4 days	2–3 h

Noteworthy, ATAC-seq has several advantages: first, the transposase method can reduce experimental time to 2–3 h to achieve DNA fragmentation using a simple enzymatic reaction, which avoids the tedious conventional DNA fragmentation, terminal repairing and adapter connection reaction [38]. On the other hand, it usually takes 2–3 days to prepare the DNase-seq and MNase-seq experiments, and 3–4 days for the FAIRE-seq experiment. Second, the simplified experimental procedure reduces the duration of sample preparation and decreases the probability of errors, which significantly improves the successful rate and repeatability of an experiment. Third, the sample size is reduced by at least 1000 times, by decreasing from 1 to 50 million cells (FAIRE-seq) and 50 million cells (DNase-seq) to as low as about 500 cells [38, 48–50]. When sample collection is challenging, this advantage is particularly prominent. Fourth, ATAC-seq can use paired-end sequencing technology to map nucleosome positioning and occupancy [51]. Paired-end sequencing can sequence both ends of the DNA fragment, making the alignment of reads mapping over repetitive regions of the genome more accurate [52].

There are also some limitations of the ATAC-seq technology. First, the Tn5 transposase simultaneously fragments and tags unprotected regions of DNA with sequencing adapters through the “cut and paste” mechanism. The adapter joints at both ends of each DNA fragment are random, which leads to a 50% probability of that the adapters at both ends of one fragment are the same, generating half unusable fragments for enrichment, amplification and sequencing [53]. Second, studies have shown that “naked” DNA without nucleosomes and transcription factors is easier to be cleaved by the Tn5 transposase [27]. Moreover, the Tn5 transposase tends to bind and cleave at transcription factor binding regions, which results in a loss of part of the transcription factor information [54, 55]. All these drawbacks make ATAC-seq difficult to detect the footprint of transcription factors, which can be used to identify potential binding motifs of transcription factors. Third, due to presence of mitochondrial DNA, data obtained by ATAC-seq inevitably contains some mitochondrial reads. Depending on the cell type, ATAC-seq data may contain 20–80% of mitochondrial sequencing reads [56].

To obtain pure nuclear genome reads and to reduce mitochondrial contamination, two methods can be used: using the cell lysis buffer without detergent [19], and using the clustered regularly interspaced short palindromic repeats (CRISPR) technology [57–59]. The CRISPR/Cas9 technology uses guide RNA (gRNA), which can target the mitochondrial chromosome [60, 61]. By adding gRNA/Cas9 mix to prepared sequencing library, gRNA can target mitochondrial ribosomal DNA and Cas9 enzyme will cleavage the fragments [58]. Compared to the original protocol, CRISPR technology results in lower mitochondrial reads, and more reads in the nuclear genome [58, 60, 62].

### Improvement of ATAC-seq

Since the ATAC-seq method was first developed, it has been improved in order to adapt broader usage in research. Single-cell ATAC-seq (scATAC-seq) provides the first insightful examination of cell-to-cell variability in chromatin organization, which can be achieved by a programmable microfluidic device or combinatorial cellular indexing scheme. The scATAC-seq can be used as a genome-wide vehicle to map chromatin accessibility in all specific cell types of an organism [50, 63–65]. Because it is still unclear exactly how many open chromatin regions exist in a single cell, and how chromatin accessibility differs between the two alleles in an individual cell, whether the scATAC-seq does capture a limited subset of open chromatin sites in single cells remains unclear [66].

Moreover, Omni-ATAC-seq is another improved ATAC-seq protocol to detect chromatin accessibility [56]. Based on the standard ATAC-seq protocol, the Omni-ATAC-seq adds a washing step using detergents after cell lysis to remove mitochondria from the transposition reaction. The Omni-ATAC-seq also uses phosphate-buffered saline (PBS) in the transposition reaction to increase the signal-to-background ratio and to reduce the background. Thus, the Omni-ATAC-seq eliminates mitochondrial interference and reduces background noise to obtain high quality data of chromatin accessibility [56]. Moreover, the standard ATAC-seq requires the transposition reaction to be performed on fresh cells, and slowly cooled cryopreserved cells, but poorly on snap-frozen cells [67]. The Omni-ATAC protocol can generate high-quality chromatin accessibility profiles from clinically relevant frozen tissues, such as brains [56].

ATAC-seq obtains the information of accessible chromatin by breaking up cells, so it cannot describe the three-dimensional structure of these accessible genomic regions. Assay for Transposase Accessible Chromatin with Visualization (ATAC-see) uses the same enzymatic methods as ATAC-seq, and adds fluorescent clusters together with DNA markers, which allows visualization of three-dimensional immobilized nuclei [68].

### Applications

Applying ATAC-seq has advanced our understanding of the machinery of gene expression regulation, such as chromatin accessibility between different samples, nucleosome positions, and genome-wide binding sites of transcription factors [23, 69, 70]. It has provided meaningful insight into revealing the landscape of chromosome accessibility, epigenetic modification of embryonic development, epigenetic mechanism of tumorigenesis, and potential disease biomarkers [61, 71–74]. Here, we focus on applications of ATAC-seq in basic research and clinical usage.

### ATAC-seq in mapping the accessible chromatin landscape

Mapping the accessible chromatin landscape can obtain information of spatial changes in chromatin structures and transcription factors associated with gene expression [59]. This information can reveal the network of relevant transcription factors, and mechanisms of chromatin structural regulation that governs gene expression programs [59]. For instance, in the human immune system, the accessible chromatin map of primary immune cells—T lymphocytes has been identified by using ATAC-seq [75]. A significant change of chromatin accessibility has been identified in regions near genes that are associated with B cell activation, especially in Systemic Lupus Erythematosus (SLE) patients [76].

In developmental biology, the lineage-specific open chromatin regions and changes have been mapped using ATAC-seq in epidermal differentiation, and in trophoblast stem cell differentiation in placenta [77, 78]. In the developing heart, transcription factor TBX20 has been identified to bind to the conserved long-range enhancer Vcan, and to co-regulate gene expression [79]. In the nervous system, induced activation of neurons leads to instantaneous changes in the chromatin structure, especially in the enhancer region [80]. Mapping the accessible chromatin landscape of the developing cerebral cortex has identified enhancers for *FGFR2* and *EOMES* as important regulatory players in cortical neurogenesis [81]. Moreover, ATAC-seq has been used to obtain landscapes of accessible chromatin of endocrine cells and germ cells [32, 82–86].

In summary, applying ATAC-seq has generated comprehensive accessible chromatin landscapes of various cell types in different tissues and organs, which has provided valuable insights into the complexity of gene transcription.

### ATAC-seq in embryonic development

Chromatin reprogramming actively occurs during early embryonic development [61]. Studies have shown that when chromatin reprogramming happens, regulatory factors that are associated with gene transcription and DNA recombination are recruited by chromatin [87, 88], and simultaneously the stability of nucleosome is altered [89]. During zygote gene activation, the activity of open chromatin is increased, in parallel with activities of *cis*-regulatory factors, which confirms that *cis*-regulatory elements play a significant role in early development [90, 91]. ATAC-seq has been used, together with the CRISP/Cas-9 technology, to detect mouse preimplantation embryos [61]. Chromatin atlas of mouse early embryos at different developmental stages have been drawn by ATAC-seq, and motifs of essential transcription factors for early development such as CTCF, NR5A2 and TEAD4 have been identified [61].

Moreover, to study embryonic genome activation, ATAC-seq has been used to detect transcriptome sequences [92]. These studies have shown that multiple copies of *DUX4* are activated by endogenous genes *KDM4E* and *ZSCAN4* that are expressed only in cleavage-stage of human embryos, which subsequently initiates transition of embryonic stem cells to 2-cells stage with totipotency [92–97].

### ATAC-seq in cancer research

ATAC-seq is highly applicable to capture the tissue-specific chromatin activity of regulatory regions in tumors [18, 75, 98–100]. In Ras-dependent oncogenesis, 3778 over-activated regulatory regions are detected by using ATAC-seq [18, 101]. Recurrent mutations in *RAD21* and *STAG2* genes, which encode the chromosome cohesion complex, have been shown to be key elements in malignancy formation in acute myeloid leukemia (AML) [98, 102–104]. Studies have shown that mutant cohesin can increase chromatin accessibility of binding sites for transcription factors such as ERG, GATA2 and RUNX1, as detected by using ATAC-seq [105–108].

Moreover, *ARID1A* mutations usually occur in many kinds of tumors, such as melanoma, glioblastoma and other human malignancies [109–113], and *ARID1B* mutations are usually found in neuroblastoma, hepatocellular carcinoma and breast invasive ductal carcinoma [114–117]. Studies have shown that mutations of the *ARID1A* and *ARID1B* complex are frequently associated with tumorigenesis via altering promoter and enhancer activities to modulate downstream gene expression [118]. During the cell neoplastic transformation, down-regulation of *ARID1A* leads to H3K27ac reduction at enhancer regions of downstream genes for *ARID1A* [119, 120]. *ARID1A* plays an important role in maintaining chromatin accessibility at enhancers. In particular, the expression of *MET* gene has been changed in *ARID1A* mutant ovarian cancer cells, while *ARID1B* deletion displays the same effect only in the context of *ARID1A* mutation, indicating an important role of *ARID1A* in ovarian cancer cells [120].

Furthermore, *p53* is a well-studied cancer suppressor gene. The protein encoded by *p53* has a role of suppressing cancer under normal circumstances, and promoting cancer development when mutations occur [121, 122]. When DNA damage occurs, p53 initiates cell apoptosis by regulating gene expression [123]. Studies have shown that p53 can bind to the promoter and enhancer of a gene to excel function [124, 125]. It has been found that *p53* has a prior binding to the enhancer in healthy fibroblasts detected using ATAC-seq [126]. When DNA damage occurs, chromatin is converted from inaccessible to accessible status, and simultaneously, *p53* gene is activated to maintain genome stability [127].

## Conclusion

ATAC-seq uses high-throughput sequencing approach to identify all active regulatory sequences in the genome using a small amount of cells. ATAC-seq has been widely used in the acquisition of open chromatin regions and transcription factor binding sites to reveal a real time profile of chromatin accessibility. It has been rapidly applied and accepted to investigate gene expression dynamics in stem cells, early embryos, and various tumors, and even to detect potential biomarkers. Taking advantage of optimization of ATAC-seq methodology to simplify the experimental procedure and to reduce the cost, ATAC-seq should soon have a broader usage in basic research and clinical diagnostics.

## Data Availability

Not applicable.

## Abbreviations

AML: Acute myeloid leukemia
ATAC-see: Assay for Transposase Accessible Chromatin with Visualization
ATAC-seq: Assay for Transposase Accessible Chromatin with high-throughput sequencing
CRISPR: Clustered regularly interspaced short palindromic repeats
DNase-seq: Deoxyribonuclease I hypersensitive sites sequencing
FAIRE-seq: Formaldehyde-Assisted Isolation of Regulatory Elements
MNase-seq: sequencing of Micrococcal Nuclease sensitive sites
PAHs: Polycyclic aromatic hydrocarbons
PBS: Phosphate-buffered saline
SLE: Systemic Lupus Erythematosus
